# Ultrasonic Synthesis, Molecular Structure and Mechanistic Study of 1,3-Dipolar Cycloaddition Reaction of 1-Alkynylpyridinium-3-olate and Acetylene Derivatives

**DOI:** 10.3390/molecules21070848

**Published:** 2016-06-29

**Authors:** Asmaa Aboelnaga, Mohamed Hagar, Saied M. Soliman

**Affiliations:** 1 Department of Chemistry, Faculty of Science, Taibah University, Yanbu branch, Yanbu 00000, Saudi Arabia; asmaa_aboelnaga77@yahoo.com; 2 Department of Chemistry, Faculty of Women for Arts, Science and Education, Ain Shams University, Heliopolis, Cairo 11757, Egypt; 3 Department of Chemistry, College of Science & Arts, King Abdulaziz University, P.O. Box 344, Rabigh 21911, Saudi Arabia; saied1soliman@yahoo.com; 4 Department of Chemistry, Faculty of Science, Alexandria University, P.O. Box 426, Ibrahimia, Alexandria 21321, Egypt

**Keywords:** ultrasonic synthesis, 1,3-dipolar cycloaddition, molecular structure, reaction mechanism

## Abstract

Regioselectively, ethyl propiolate reacted with 1-(propergyl)-pyridinium-3-olate to give two regioisomers; ethyl 4-oxo-8-(prop-2-ynyl)-8-aza-bicyclo(3.2.1)octa-2,6-diene-6-carboxylate **4**, ethyl 2-oxo-8-(prop-2-ynyl)-8-aza-bicyclo(3.2.1)octa-3,6-diene-6-carboxylate **5** as well as ethyl 2,6-dihydro-6-(prop-2-ynyl)furo(2,3-c)pyridine-3-carboxylate **6**. The obtained compounds were identified by their spectral (IR, mass and NMR) data. Moreover, DFT quantum chemical calculations were used to study the mechanism of the cycloaddition reaction. The regioselectivity was explained using transition state calculations, where the calculations agreed with the formation of products **4** and **5** in almost the same ratio. The reaction was also extended for diphenylaceylene as dipolarophile to give only two products instead of three.

## 1. Introduction

Nowadays, improving the traditional methods for organic synthesis is an important issue. Ultrasound is widely used to avoid using expensive reagents, highly acidic solutions, long reaction times and high temperatures as well as to improve reaction yields and incompatibility with other functional groups [1].

The use of ultrasound irradiation in the organic synthesis offers many benefits, which include milder reaction conditions, decrease in the reaction times, and increase in selectivity, thus enhancing the purity and reaction efficiency [2,3,4]. Ultrasound irradiation has been used for the synthesis of simple heterocyles [5,6,7], various ionic liquids [8], and poly functionalized heterocyclic compounds [9] and has been reported to promote the reaction rates and yields. One of the fascinating chemical reactions of pyridin-3-ol is the exhibition of 1,3-dipolar characters.

**Scheme 1:**
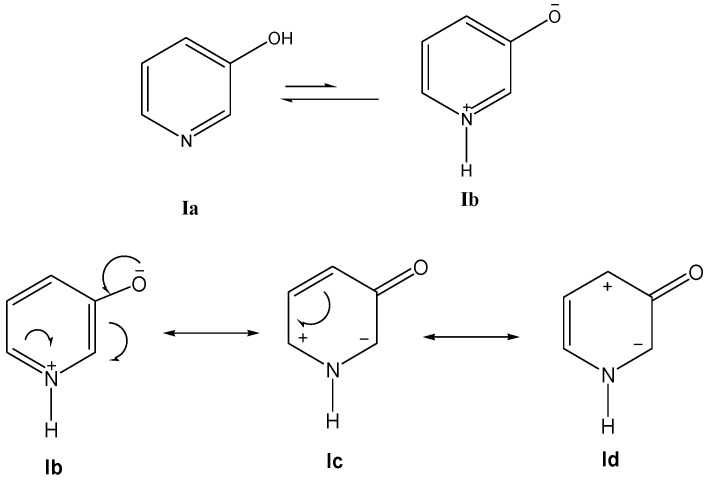
Resonating structures of 1-(propergyl)-pyridinium-3-olate.

Based on the spectroscopic data [10,11,12,13,14], pyridin-3-ol **Ia** in polar solvents exists to a considerable extent in the unstable mesomeric form, pyridinium-3-olate **Ib**, via prototropic rearrangement [15] (which is being considered as a resonance hybrid between the indicated canonical structures) (Scheme 1).

The aim of this work focused on the use of ultrasound to promote 1,3-dipolar cycloaddition reaction of the, 1-(propergyl)-pyridinium-3-olate **1** with acetylene derivatives.

## 2. Results and Discussion

### 2.1. Chemistry

**Scheme 2:**
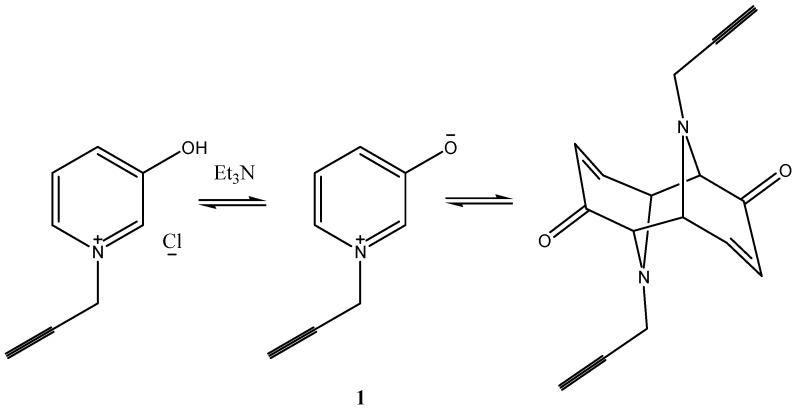
Formation of 1-(propergyl)-pyridinium-3-olate and its dimerization.

Treatment of pyridin-3-ol with propargylchloride in ethanol afforded directly the corresponding quaternary pyridinium salt as a pale yellow crystalline solid. Upon treatment of the salt with triethylamine, the mixture was subjected to ultrasound at 40 °C for 1 h. The reaction yielded a product with molecular formula of C_16_H_14_O_2_N_2_ The product was assigned as the dimeric form of the salt formed via the intermediate pyridinium-3-olate **1** (Scheme 2). The IR spectrum of the dimer revealed the characteristic absorption bands of conjugated carbonyl groups at 1734 and 1690 cm^−1^. The ^1^H-NMR spectrum revealed a singlet signal at δ = 2.05 for two acetylenic-H and 2 AB system at δ = 4.23 ppm for four methine protons along with the doublets of olefenic and the multiplet of cycloalkanes protons.

#### The Reaction of Pyridinium-3-olate (**1**) with Ethyl Propiolate (**2**) as 2π-1,3-Dipolarophiles

**Scheme 3:**
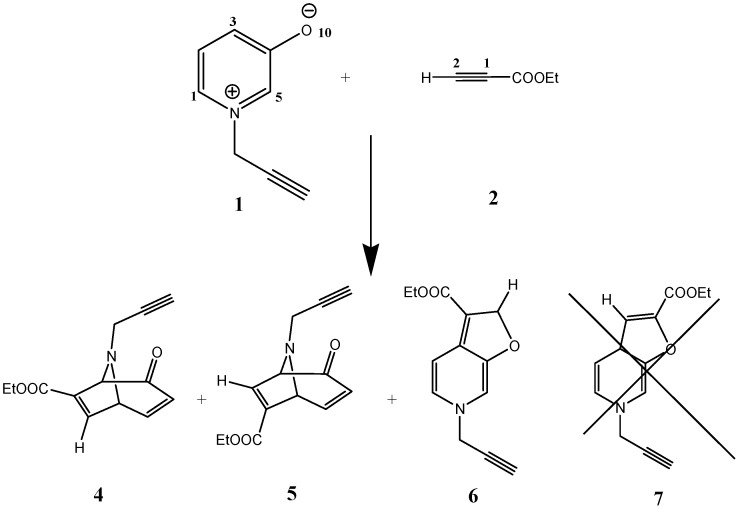
Cycloaddition of ethyl propiolate with 1-(propergyl)-pyridinium-3-olate.

The cycloaddition reaction between 1-(propergyl)pyridinium-3-olate 1(4π-1,3-dipole across the 2,6-posions as well as O-C4 region of the pyridine ring) and asymmetric 2π-acetylene 1,3-dipolarophiles, ethyl propiolate **2**, proceeded effectively in a regioselective manner to give two regioisomers, ethyl 4-oxo-8-(prop-2-ynyl)-8-aza-bicyclo(3.2.1)octa-2,6-diene-6-carboxylate **4** and ethyl 2-oxo-8-(prop-2-ynyl)-8-aza-bicyclo(3.2.1)octa-3,6-diene-6-carboxylate **5**, as the major products (32% and 27%, respectively) and ethyl 2,6-dihydro-6-(prop-2-ynyl)furo(2,3-c)pyridine-3-carboxylate **6** as a minor product (11%). It is worth noting that the cycloaddition product **7** is not obtained (Scheme 3).

### 2.2. Computational Details

Full geometry optimizations were carried out for the reactants, transition states (TSs) and cycloaddition products (CAs) using the B3LYP [16,17]. DFT function and the 6-31G (d, p) basis set. All calculations were carried out with GAUSSIAN 03 [18]. The stationary points were characterized by frequency calculations in order to verify that minima and transition states have zero and one imaginary frequency, respectively. Each TS gave one negative vibrational mode corresponding to the motion involving the formation of the newly forming C-C bonds. The vibrational modes were assigned appropriately by means of visual inspection and animation using the Gaussview software [19]. The reported total energies include zero point energy (ZPE) corrections are given at 298.15 K. Analysis of the frontier molecular orbital (FMO) interactions, global electrophilicity index (ω), was calculated following the expression [20,21,22,23], ω = (μ^2^/2η), where μ is the electronic chemical potential, μ = (E_H_ + E_L_)/2, where E_H_ and E_L_ are FMO energies and η is the chemical hardness, η = (E_L_ − E_H_). The nucleophilicity index, *N* [21], which is defined as *N* = E_H(Nu)_ − E_H(TCE)_, where tetracyanoethylene (TCE) is chosen as the reference [24,25]. The atomic electronic population and DFT-based reactivity indices were computed using natural population analysis (NPA) [26]. The local electrophilicity *ω_k_* and nucleophilicity *N_k_* indices of atom k are obtained by the help of Fukui index (f_k_) using equations:

(1) $$\omega_{k}=\omega f_{k}^{+}=\omega [Q_{k}(N+1)-Q_{k}(N)]$$

(2) $$N_{k}=Nf_{k}^{-}=\omega [Q_{k}(N)-Q_{k}(N-1)]$$

where Q_k_(*N*), Q_k_(*N* + 1), Q_k_(*N* − 1) are the gross electronic population of site k in neutral, anionic, and cationic systems, respectively [27]. The optimized structures of the reactant molecules are shown in Figure S1 (Supplementary Materials).

**Figure 1:**
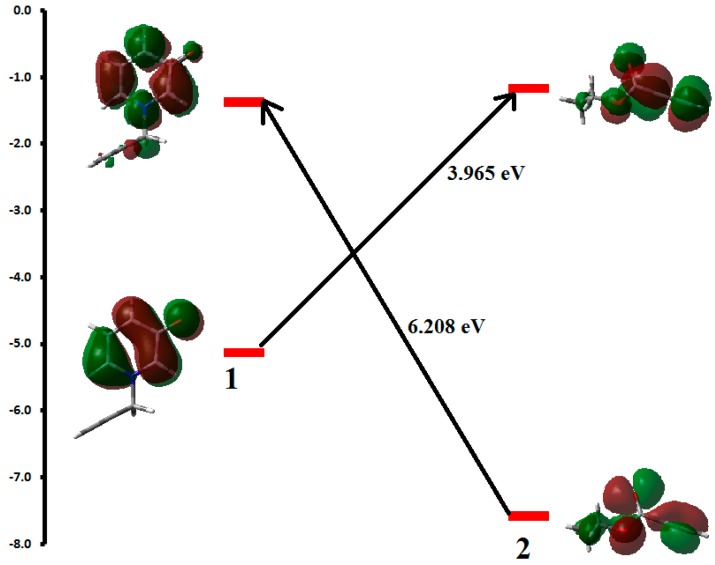
Schematic representation of the possible interactions between the FMOs.

We used DFT calculations in order to explain the regioselectivity of the studied reaction system. The reaction goes regioselectively to form the products **4**–**6** in different yields while **7** is not formed. Table 1 shows the values of the FMO energies (eV), the electronic chemical potentials (μ), the global electrophilicity (ω) and nucleophilicity (*N*) of the reactants. Figure 1 presents a schematic representation of the possible interactions between the FMOs (HOMO of **1**-LUMO of **2**) and (HOMO of **2**-LUMO of **1**). Table 1 and Figure 1 show that the gap between HOMO of **1** and LUMO of **2** is smaller (3.965 eV) than the gap between HOMO of **2** and LUMO of **1** (6.208 eV). It implies that the main interaction occurs between the HOMO of **1** and the LUMO of **2**

Moreover, the electronic chemical potential of **1** (−3.2579 eV) is higher than that of **2** (−4.3793 eV) indicating that charge transfer will take place from the reactant **1** to **2**, which is in agreement with the FMO analysis. The amount of charge transfer from **1** to **2** is calculated to be 0.1427 e, 0.1326 e and 0.2172 e for products **4**, **5** and **6**, respectively. These results indicate that the studied cycloaddition reactions have polar character. The amount of charge transfer is the highest for TS of **6** so; it has the highest polar cycloaddition (CA) reaction. At the transition state structures, the reactant **1** fragment has positive charge while fragment of reactant **2** have negative charge which confirm that **1** is the nucleophile while **2** is the electrophile in these CA reactions.

Domingo et al. classified electrophiles based on the electrophilicity index as strong (ω > 1.50 eV), moderate (1.50 > ω > 0.80 eV) and marginal (ω < 0.80 eV). In addition, nucleophiles are classified as strong (*N* > 3.00 eV), moderate (3.00 > *N* > 2.00 eV) and marginal (*N* < 2.00 eV) [28,29,30]. According to the classification, **1** is a strong nucleophile and moderate electrophile, whereas **2** is moderate electrophile and marginal nucleophile. Based on the values of nucleohilicity index (*N*) listed in Table 1, **1** has higher nucleophilicity (3.9830 eV) than **2** (1.5345 eV). On the other hand, the electrophlicity index (ω) of **1** (1.4117 eV) is lower than **2** (1.4951 eV). As a result, **1** is the nucleophile while **2** is the electrophile in this reaction, in agreement with the previous analyses.

**Scheme 4:**
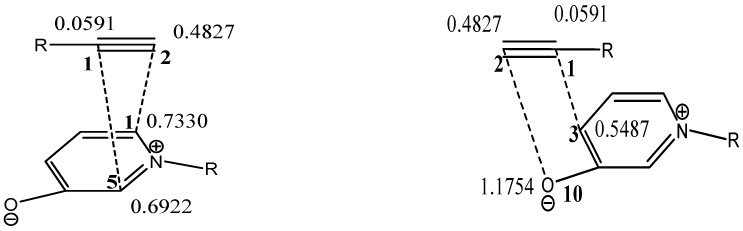
Illustration of the favorable interactions using local electrophilicity indices.

The local electrophilicity indices ω*_k_* and *N_k_* of atom k were used to explain the regioselectivity of the studied CA reaction. The values of the Fukui indices ($f_{k}^{+}$ and $f_{k}^{-}$) and local electrophilicity indices (ω*_k_* and *N_k_*) are reported in Table 2. The atom numbering Scheme of the reactants are given in Figure S1 (Supplementary Materials). For better visualization, we have depicted these interactions in Scheme 4. It is slightly more favorable if C1 and C2 of reactant **2** attack on C5 and C1 of **1**, respectively rather than the C1 and C5 of **1**, respectively. Hence, one could predict that **4** will be formed slightly more than **5**. Experimentally, we observed the formation of product **6** while **7** was not formed at all. Based on the magnitude of local descriptors shown in Scheme 4, it is clear that in the [2 + 3] cycloaddition reaction, the C1 and C2 of reactant **2** favor the attack on C3 and O10 of **1**, giving product **6** as the only furan product.

**Figure 2:**
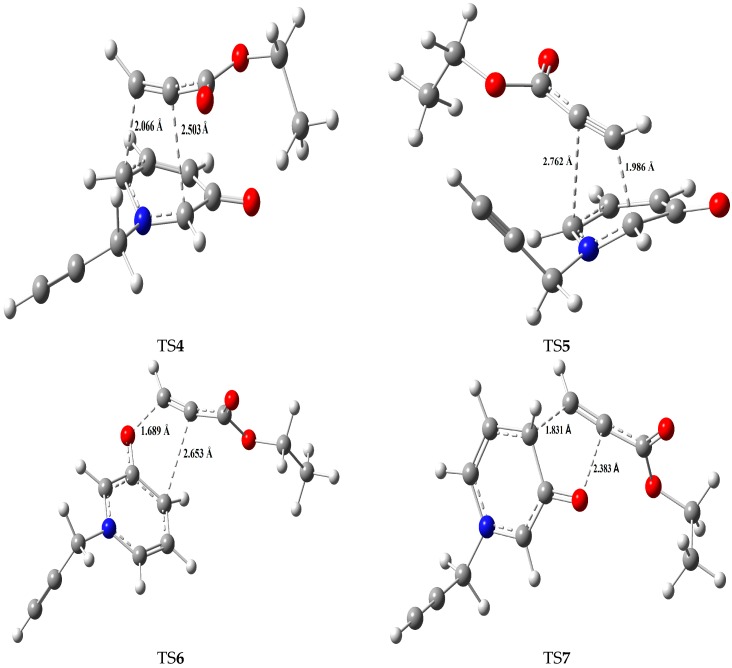
The four transition state structures of the studied CA reaction.

**Figure 3:**
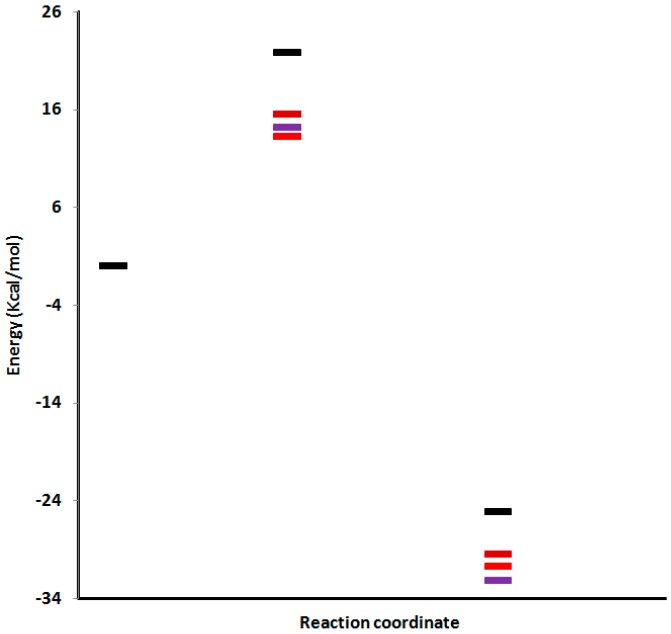
Energy profiles, in kcal/mol for the two pathways of the CA reactions (red; **4**, violet **5**, brown **6** and black **7**).

In order to explain the formation of **4** and **5** as the major products, **6** as a minor product, and no formation of **7**, we performed transition state calculations for the four regioisomeric pathways. The four transitions states of the reactions leading to the formation of products **4**, **5**, **6** and **7** will be abbreviated TS4, Ts5, Ts6 and TS7, respectively. The geometries of these four TSs are given in Figure 2 together with the newly forming bond lengths. Table 3 reports the energies (a.u.) and relative energies (kcal/mol). The potential energy surfaces (PESs) corresponding to all the reaction channels, are illustrated in Figure 3. Based on the calculated energy difference between the product and reactants, all products are stable thermodynamically where the most stable product is **5**. On the other hand, the calculated activation energies of the different reaction pathways between **1** and **2** showed that adduct **4** is the most favored kinetically in comparison with the other approaches. The small energy difference (0.984 Kcal/mol) between the two TSs of adducts **4** and **5** indicated that why these two products are formed in almost the same proportions. The high activation energy of product **7** TS (21.849 Kcal/mol) suggested that **7** could not be formed while **6** is formed although in lower amount than the others (**4** and **5**). These results agree with what we observed experimentally.

**Scheme 5:**
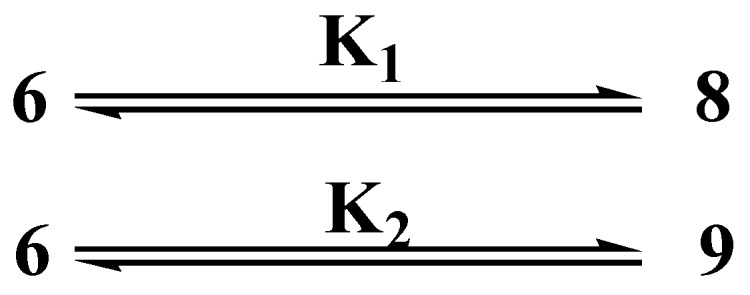
The equilibrium constants among the proton transfer structures of adduct 6.

**Figure 4:**
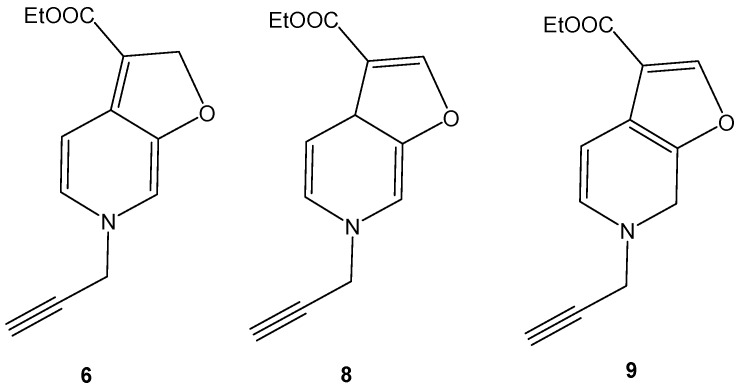
The possible regioisomers of **6**.

It is worth noting that product **6** could undergo rearrangement via proton transfer leading to two other possible structures (**8** and **9**) shown in Figure 4. These products could not be easily differentiated experimentally using routine NMR measurements as the H and C atoms of the three adducts have very close chemical shifts. Form this point of view, theoretical calculations on the three suggested products of **6** were performed in order to predict the most stable adduct based on the energy analysis of these compounds. The energies and thermodynamic parameters of the equilibrium reactions shown in Scheme 5 are given in Table 4. The relative energy and relative Gibbs free energy are given in Kcal/mol.

It is found that **6** has the lowest energy and hence the most stable form. The rearrangement product **6** is more stable by 5.3823 and 23.4370 kcal/mol than **9** and **8**, respectively. In addition, the negative Gibbs free energy change indicated that the rearrangement process from **8** and **9** to **6** is spontaneous. Using the relation ΔG = −RTlnK, the equilibrium constants were obtained where ΔG is the difference between the Gibbs free energy of **8** or **9** with respect to the most stable one (**6**). Using the equilibrium constants obtained from such calculations, the ratio of the three products **8**, **9** and **6** were obtained. The results showed that **6** has 99.98% and it is only one that could exist.

### 2.3. NMR Spectra

**Figure 5:**
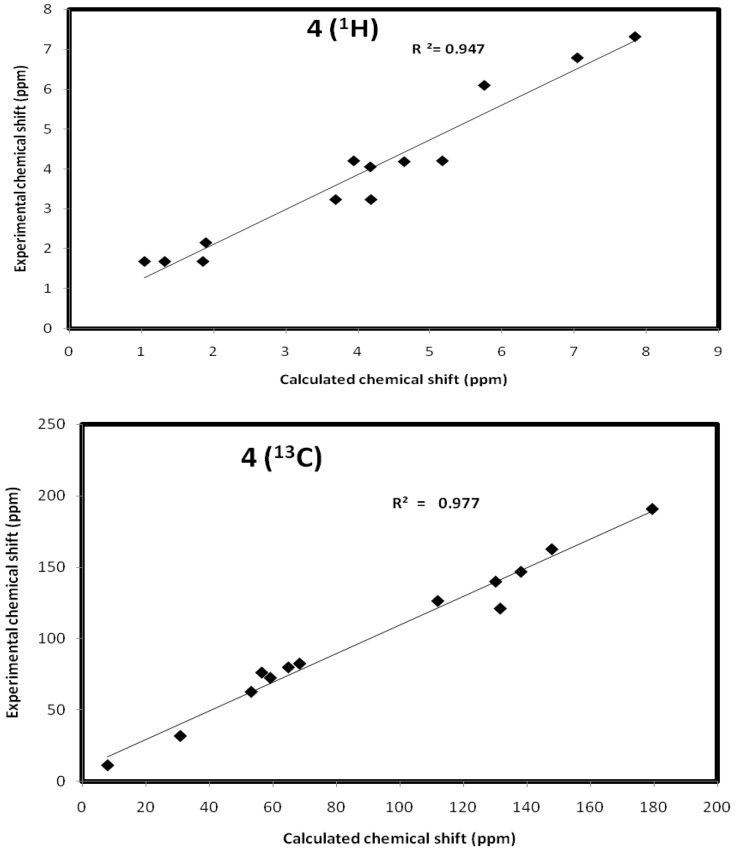
The correlation graphs between the calculated and experimental chemical shifts compound **4**.

**Figure 6:**
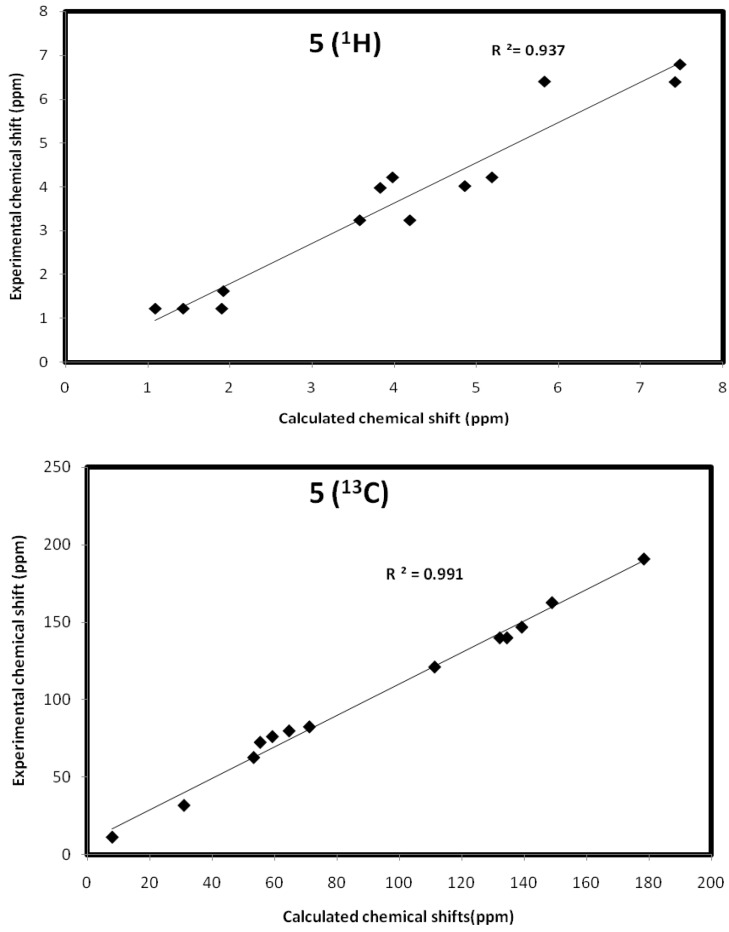
The correlation graphs between the calculated and experimental chemical shifts compound **5**.

**Figure 7:**
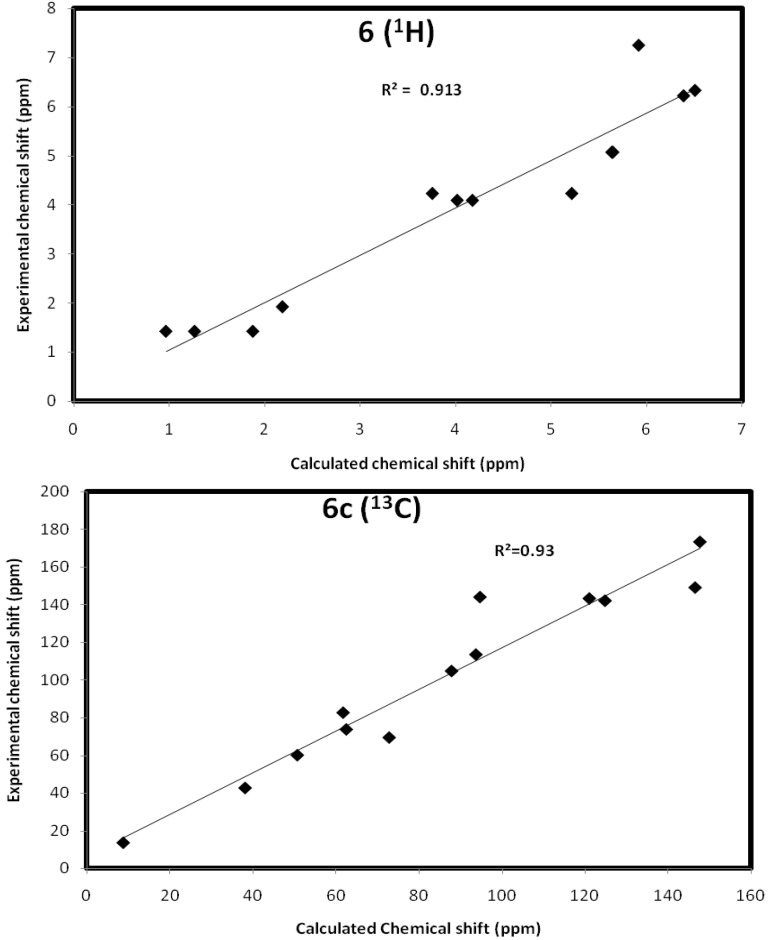
The correlation graphs between the calculated and experimental chemical shifts compound **6**.

The values of isotropic magnetic shielding (IMS) are calculated by the GIAO approach at the 6-31G (d, p) level that were used to predict the ^13^C- and ^1^H-NMR chemical shifts (δ_calc_) for the studied compound, and the results were correlated with the experimental NMR data (δ_exp_) in CDCl_3_ solvent. Figure 5, Figure 6 and Figure 7 and Table 5, Table 6 and Table 7 showed the correlation between the experimental and theoretical ^13^C- and ^1^H-NMR chemical shifts of the studied compounds. According to the results, the calculated chemical shifts were in good agreement with the experimental findings. The correlation coefficients for ^13^C- and ^1^H-NMR chemical shifts are up to 0.991 and 0.947, respectively.

**Scheme 6:**
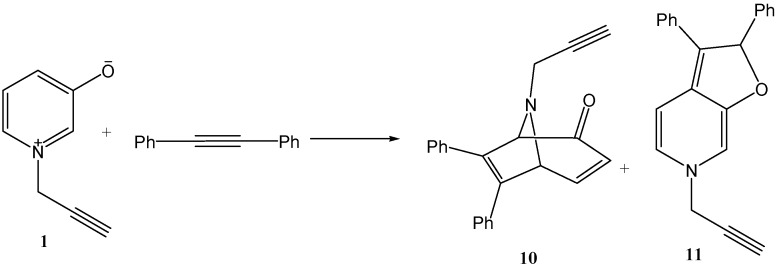
Cycloaddition of diphenylacetylene with 1-(propergyl)-pyridinium-3-olate.

On the other hand, this reaction was extended for the cycloaddition reaction of 1-(propergyl)pyridinium-3-olate **1** with diphenylacetylene as symmetric dipolarophile to afford only two products, 6,7-diphenyl-8-(prop-2-ynyl)-8-aza-bicyclo(3.2.1)octa-3,6-dien-2-one **10** as major product and 2,6-dihydro-2,3-diphenyl-6-(prop-2-ynyl)furo(2,3-c)pyridine **11** as a minor product (Scheme 6).

## 3. Materials and Methods

*1-(propergyl)-pyridinium-3-olate* (**1**): A mixture of pyridin-3-ol (1.9 g, 0.02 mol) and propargyl chloride (1.45 g, 0.02 mol) in absolute alcohol (30 mL) was heated under reflux in water bath while stirring for 3 h. The reaction mixture was allowed to cool until room temperature; the solvent was removed under reduced pressure. The yellow residue obtained was recrystallized from ethanol to give **1** as pale yellow crystals yield 88%, mp 98–100 °C. IR: ν 3401–3250 (broad, OH), 2364 (acetylenic bond), 1571, 1241 cm^−1^ (ring vibrations). ^1^H-NMR (CDCl_3_): δ = 2.05 (s, 1H, acetylenic-H), 3.25 (s, 1H, OH), 3.52 (s, 1H, H-2), 3.75 (d, 1H, H-5), 5.2 (s, 1H, CH_2_), 5.92 (dd, 1H, H-3), 6.48 (d, 1H, H-4). Anal. Calcd for C_8_H_8_NOCl (169.6): MS (EI): *m*/*z* = 133 [M^+^ − HCl], base peak at *m*/*z* = 95 [M^+^ − Cl-CH_2_CCH].

*Reaction of 1-(propergyl)-pyridinium-3-olate (**1**) with ethyl propiolate* (**2**): To a mixture of 3-hydroxy prop-2-yne-1-yl-pyridinium chloride (1.69 g, 0.01 mol) and ethyl propiolate (2 mL, 0.026 mol) in the presence of hydroquinone (0.01 g), triethylamine (1 mL) was added dropwise. The reaction mixture was heated in ultrasonic bath, for 6 h at 40 °C. At the end of the reaction, the brown residue obtained was triturated with distilled water and then extracted with chloroform that was separated, dried over anhydrous sodium sulfate, filtered off, and evaporated under reduced pressure to give a brown sticky residue, which gave three spots on TLC. The residue obtained was eluted through a column of alumina with a mixture of ethyl acetate-light petroleum (1:2). Three products **4**–**6** were obtained in a combined yield of 80% (1.84 g).

*Ethyl 4-oxo-8-(prop-2-ynyl)-8-aza-bicyclo(3.2.1)octa-2,6-diene-6-carboxylate* (**4**): Yellow crystals (0.61 g, 32%), mp 176–177 °C, IR ν = 2929 (CH), 1698 (conjugated CO), 1735 cm^−1^ (CO ester), C_13_H_13_NO_3_ (231.25), [M^+^] at *m*/*z* = 231, base peak at *m*/*z* = 80.

*Ethyl 2-oxo-8-(prop-2-ynyl)-8-aza-bicyclo[3.2.1]octa-3,6-diene-6-carboxylate* (**5**): Orange crystals (0.52 g, 27.5%), mp 180–182 °C, IR ν = 2931 (CH), 1686 (conjugated CO), 1726 cm^−1^ (CO ester), C_13_H_13_NO_3_ (231.25), [M^+^] at *m*/*z* = 231, base peak at *m*/*z* = 80.

*Ethyl 2,6-dihydro-6-(prop-2-ynyl)furo(2,3-c)pyridine-3-carboxylate* (**6**): Yellow oil (0.20 g, 11.0%), IR ν = 2924 (CH), 1723 (CO ester), 1616 (C=C-N), 1348 cm^−1^ (furan ring). C_13_H_13_NO_3_ (231.24), [M^+^] at *m*/*z* = 231, base peak at *m*/*z* = 180.

### 3.1. Reaction of 3-Hydroxy-1-prop-2-yne-1-ylpyridinium Chloride with Diphenyl Acetylene

To a mixture of 1-(propergyl)-pyridinium-3-olate (**1**) (1.69 g, 0.01 mol) and diphenyl acetylene (3.56 mL, 0.02 mol) in the presence of hydroquinone (0.01 g), triethylamine (1 mL) was added dropwise. The reaction mixture was heated in ultrasonic bath, for 6 h at 40 °C. At the end of the reaction, the brown residue obtained was triturated with distilled water and then extracted with chloroform that was separated, dried over anhydrous sodium sulfate, filtered off, and evaporated under reduced pressure to give a brown sticky residue. The residue obtained was eluted through a column of alumina with a mixture of chloroform–light petroleum (3:1). The solvent was removed under reduced pressure to give two products, **10**–**11** (2.54 g, overall yield 82%).

*6,7-Diphenyl-8-(prop-2-ynyl)-8-aza-bicyclo(3.2.1)octa-3,6-dien-2-one* (**10**): A brown oil, (1.52 g , 60%), IR ν = 2920 (CH), 1690 cm^−1^ (conjugated CO). C_22_H_17_NO (311.37), [M^+^] at *m*/*z* = 311 a.m.u., base peak at *m*/*z* = 95 a.m.u ^1^H-NMR (DMSO) at δ = 2.18 (s, 1H, acetylenic-H); 4.05 (s, 2H, CH_2_); 4.93 (d, *J*_4,5_ = 5 Hz, 1H, H-5); 5.22 (s, 1H, H-1); 6.00 (d, *J*_3,4_ = 5 Hz, 1H, H-3); 6.58 (dd, *J*_4,5_ = 5 Hz, *J*_4,3_ = 5 Hz, 1H, H-4); 7.20–7.91 (m, 10H, arom-H).

*2,6-Dihydro-2,3-diphenyl-6-(prop-2-ynyl)furo(2,3-c)pyridine* (**11**): orange oil, (0.42 g, 14%), IR ν = 2924 (CH), 1628–1348 (furan ring), 1525 cm^−1^ (C=C-N). C_22_H_17_NO (311.37) C (84.84%), H (5.50%), N (4.50%) required C (84.65%), H (5.33%), N (4.42%). ^1^H-NMR (DMSO) at δ = 2.1 (s, 1H, acetylenic-H); 3.55 (s, 2H, CH_2_ methylene-H); 4.92(s, 1H, H-1); 5.2(d, 1H, H-2); 6.35 (d, 1H, H-3); 7.90–8.07 (m, 10H, arom-H and 1H, H-4).

## 4. Conclusions

The cycloaddition reaction between 1-(propergyl)pyridinium-3-olate **1** with either symmetric or asymmetric 2π-acetylene 1,3-dipolarophiles proceeded effectively in a regioselective manner to give bicyclic compounds (8-(prop-2-ynyl)-8-aza-bicyclo(3.2.1)octa-3,6-diene derivative) and 2,6-dihydro-6-(prop-2-ynyl)furo(2,3-c)pyridine derivative. The results of the DFT calculations showed that in the cycloaddition reaction between 1-(propergyl)pyridinium-3-olate **1** and ethyl propiolate **2**, the reactant **1** is the nucleophile while **2** is the electrophile. The global and local reactivity descriptors were used to study the mechanism of the studied CA reaction. Transition state calculations were used to explain the regioselectivity of the CA reaction. Product **7** could not be formed as it has the highest activation energy. Products **4**–**6** have lower activation energy where **4** and **5** have very close energy barrier so they are formed almost in the same ratio. Product **6** has higher activation energy; hence, it is formed in a lower amount than the others.

## Figures and Tables

**Table 1:** The FMO energies (E), electronic chemical potential (μ), nucleophilicty (N) and electrophilicity (ω) indices for the reactants.

Reactant	E_H_	E_L_	Μ	ω	N
**1**	−5.1376	−1.3783	−3.2579	1.4117	3.9830
**2**	−7.5860	−1.1725	−4.3793	1.4951	1.5345

**Table 2:** Electrophilic and nucleophilic Fukui indices and local electrophilicities for the reactive atoms of **1** and **6**.

Reactant	1					2	
Parameter	C1	C5	C3	O10	Parameter	C1	C2
$f_{k}^{+}$	0.1840	0.1738	0.1378	0.2951	$f_{k}^{+}$	−0.0661	−0.2783
$f_{k}^{-}$	0.1044	0.1977	0.1761	0.0936	$f_{k}^{-}$	−0.0395	−0.3229
*N_k_*	0.7330	0.6922	0.5487	1.1754	ω*_k_*	0.0591	0.4827

**Table 3:** Energies and relative energies (∆E) of the reactants, transition states, and products.

System	E (a.u.)	∆E (Kcal/mol) ^a^	∆G (Kcal/mol) ^a^
**1**	−438.804		
**2**	−344.429		
TS**4**	−783.212	13.213	26.498
TS**5**	−783.210	14.197	27.013
TS**6**	−783.208	15.539	27.231
TS**7**	−783.198	21.849	34.320
**4**	−783.282	−30.691	−16.514
**5**	−783.284	−32.179	−18.097
**6**	−783.280	−29.502	−16.561
**7**	−783.273	−25.174	−11.936

^a^ The energies of the TSs and products are referred to the sum (E**_1_** + E**_2_**).

**Table 4:** The energies, thermodynamic parameters and rate constants (k) of the studied reactants.

Energies	6	8	9
E (a.u)	−783.5544	−783.5158	−783.5455
ZPVE (a.u)	0.2371	0.2359	0.2368
E_corr_ (a.u)	−783.3172	−783.2799	−783.3087
∆E (Kcal/mol)	0.0000	−23.4370	−5.3823
H (a.u)	−783.3003	−783.2628	−783.2916
S (Cal/Mol·K)	130.8760	133.5250	131.7610
G (a.u)	−783.3625	−783.3262	−783.3542
∆G (Kcal/mol)	0.0000	−22.7472	−5.2121
K		3.69 × 1016	6.25 × 103

**Table 5:** Calculated and experimental NMR chemical shifts of compound **4**.

Atom	δ_calc_	δ_exp_	Atom	δ_calc_	δ_exp_
1 C	56.47	76.21	18 H	4.64	4.19
2 C	130.20	139.98	19 H	7.04	6.79
3 C	111.94	126.40	20 H	5.75	6.10
4 C	179.58	190.89	21 H	4.17	4.06
5 C	68.39	82.50	22 H	7.84	7.32
7 C	131.65	121.11	23 H	1.04	1.69
8 C	138.18	146.87	24 H	1.32	1.69
9 C	147.91	162.71	25 H	1.85	1.69
12 C	7.88	11.24	26 H	5.17	4.21
13 C	53.13	62.71	27 H	3.94	4.21
14 C	30.78	31.78	28 H	3.69	3.24
15 C	64.85	79.89	29 H	4.18	3.24
16 C	59.16	72.5	30 H	1.89	2.16

**Table 6:** Calculated and experimental NMR chemical shifts of compound **5**.

Atom	δ_calc_	δ_exp_	Atom	δ_calc_	δ_exp_
1 C	62.32	72.5	18 H	4.86	4.02
2 C	134.02	139.98	19 H	7.42	6.40
3 C	126.52	121.11	20 H	5.83	6.41
4 C	184.79	190.89	21 H	3.83	3.98
5 C	76.89	82.50	22 H	7.48	6.80
7 C	96.28	139.98	23 H	1.09	1.22
8 C	129.53	146.87	24 H	1.43	1.22
9 C	169.53	162.71	25 H	1.90	1.22
12 C	13.24	11.24	26 H	5.19	4.22
13 C	64.62	62.71	27 H	3.98	4.22
14 C	32.78	31.78	28 H	3.58	3.24
15 C	72.50	79.89	29 H	4.19	3.24
16 C	68.71	76.21	30 H	1.92	1.62

**Table 7:** Calculated and experimental NMR chemical shifts of compound **6**.

Atom	δ_calc_	δ_exp_	Atom	δ_calc_	δ_exp_
1 C	93.78	113.56	18 H	6.51	6.33
2 C	121.1	143.35	19 H	6.39	6.22
3 C	94.75	144.09	20 H	5.92	7.25
4 C	146.6	149.14	21 H	4.18	4.09
5 C	124.88	142.15	22 H	4.02	4.09
7 C	38.14	42.7	23 H	2.19	1.92
8 C	62.53	73.8	24 H	5.65	5.07
9 C	61.77	82.7	25 H	5.64	5.07
12 C	72.87	69.5	26 H	0.97	1.42
13 C	87.91	104.82	27 H	1.27	1.42
14 C	147.75	173.4	28 H	1.88	1.42
15 C	8.78	13.6	29 H	5.22	4.23
16 C	50.73	60.1	30 H	3.76	4.23

## Supplementary Materials

Supplementary materials can be accessed at: http://www.mdpi.com/1420-3049/21/7/848/s1.
