# Supplementary material for: Ultrasonic Synthesis, Molecular Structure and Mechanistic Study of 1,3-Dipolar Cycloaddition Reaction of 1-Alkynylpyridinium-3-olate and Acetylene Derivatives
Source: Molecules. 2016 Jun 29;21(7):848. doi: 10.3390/molecules21070848 (PMC6274580; doi:10.3390/molecules21070848)
Supplement: Supplementary file 1 [file molecules-21-00848-s001.pdf]

Supplementary Materials: Ultrasonic Synthesis, Molecular Structure and Mechanistic Study of 1,3-Dipolar Cycloaddition Reaction of 1-Alkynylpyridinium-3-olate and Acetylene Derivatives

AsmaaAbolnaga, Mohamed Hagar and Saied M. Soliman

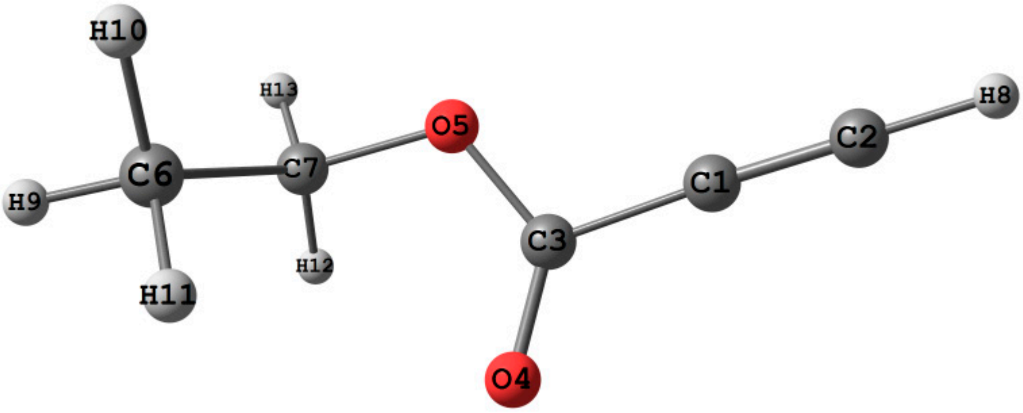

1

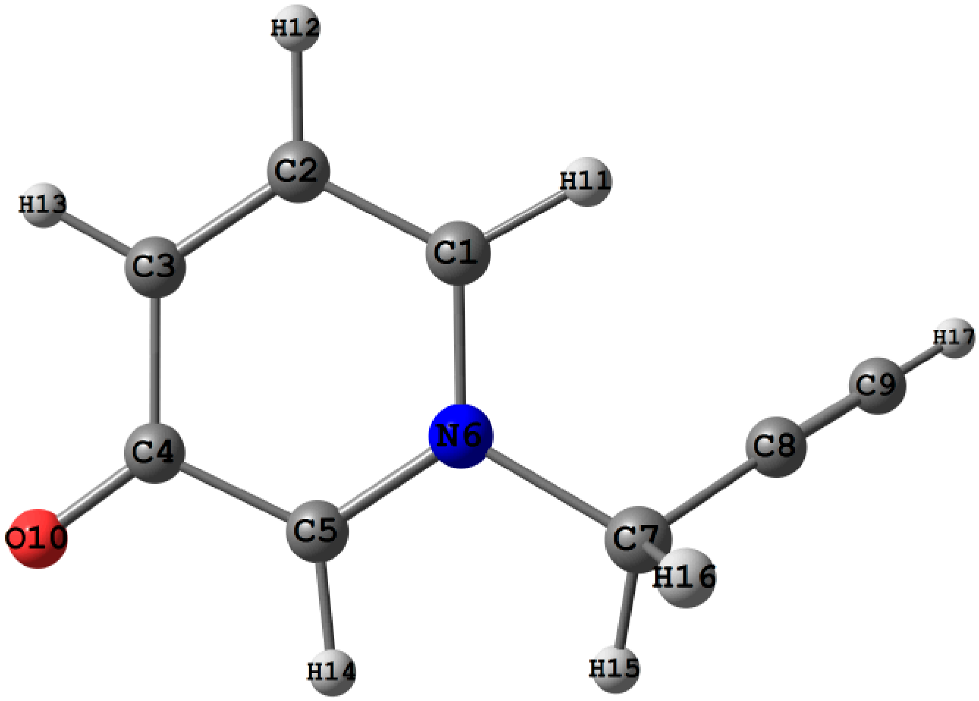

2

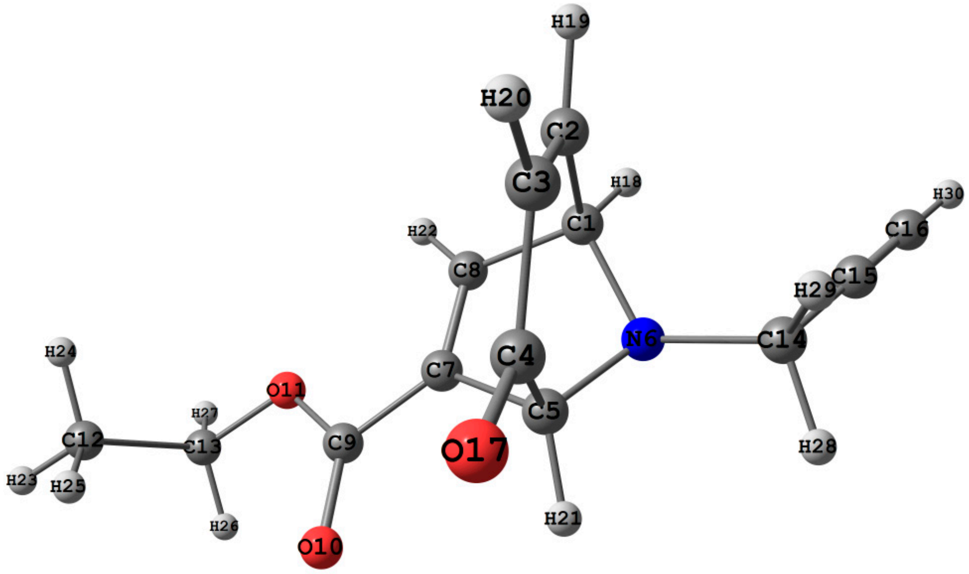
H19
H20
C2
C3
H18
H30
C1
C16
H22
C8
C15
H29
N6
C14
H24
C4
C7
O11
H27
C5
C12
C13
C9
O17
H28
H23
H25
H26
H21
O10

4

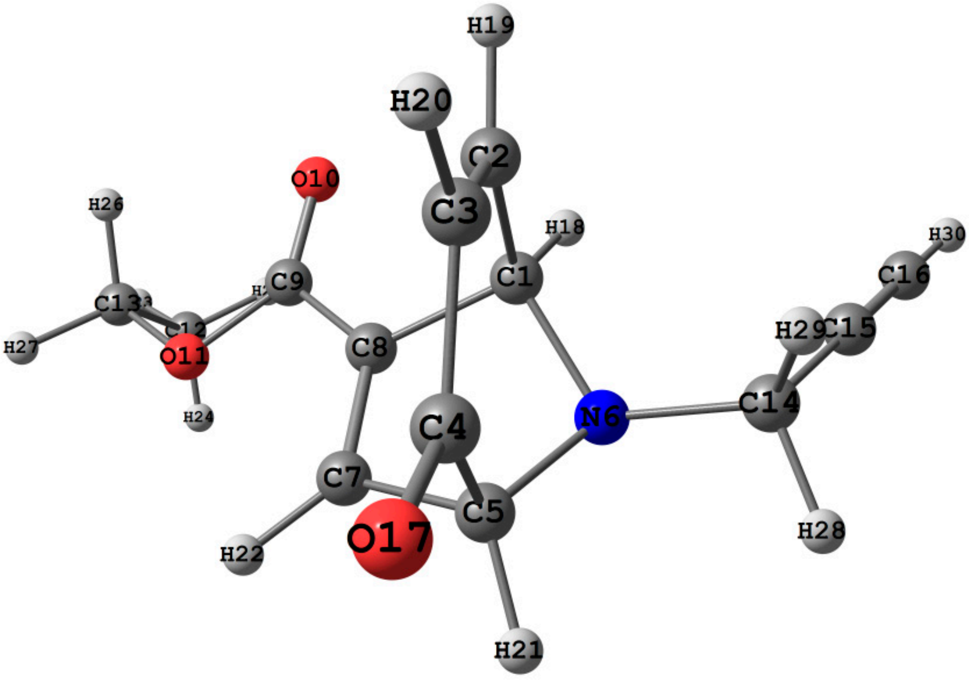
H19
H20
C2
O10
C3
H26
H18
H30
C9
C1
C16
C13
C12
H29
C15
H27
C8
O11
N6
C14
H24
C4
C7
C5
O17
H22
H28
H21

5

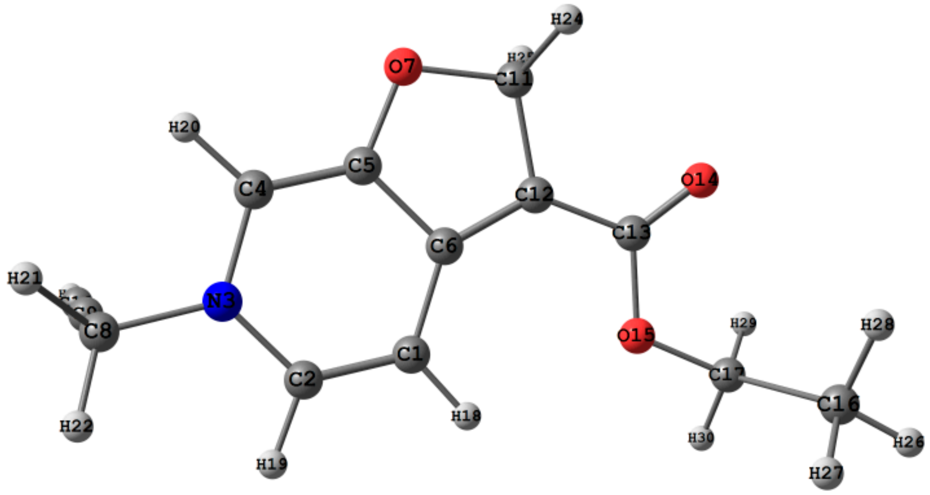
H24
O7
C11
H20
C5
C4
C12
O14
C6
C13
H21
N3
C8
H29
H28
O15
C1
C2
C17
C16
H18
H22
H30
H26
H19
H27

6

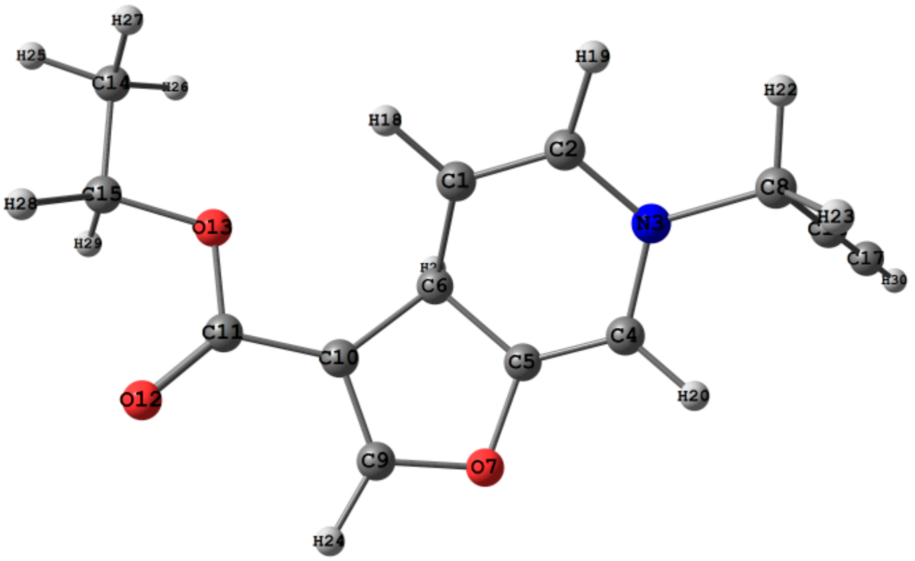

8

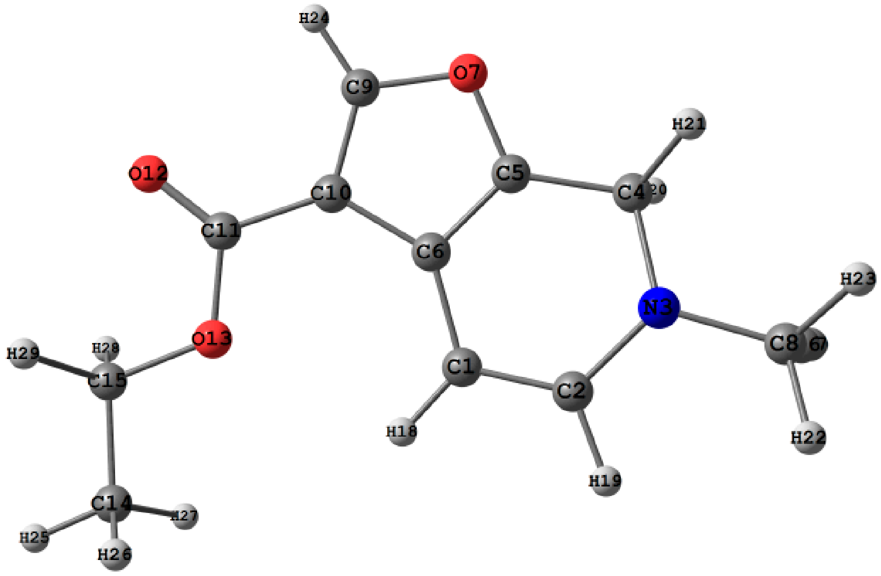

9

Figure S1. The optimized structure of the reactants.

Reactant # 1

C	−1.897000000	−0.148590000	0.086832000
C	−3.03751500	−0.50152500	0.266665000
C	−0.546737000	0.340240000	−0.118326000
O	−0.238526000	1.512910000	−0.073140000
O	0.298100000	−0.679431000	−0.365772000
C	2.437918000	−0.184631000	0.729626000
C	1.687733000	−0.318756000	−0.585981000
H	−4.044831000	−0.812962000	0.425874000
H	3.497298000	0.008607000	0.531647000
H	2.359864000	−1.103537000	1.317446000
H	2.041948000	0.647626000	1.316181000
H	1.722610000	0.609714000	−1.160323000
H	2.080123000	−1.137708000	−1.192419000

Reactant # 2

C	0.331636000	1.131793000	0.288948000
C	−0.902033000	1.762697000	0.108643000
C	−2.056684000	1.040155000	−0.102323000
C	−2.053251000	−0.413583000	−0.149350000
C	−0.729572000	−0.977774000	0.046332000
N	0.368429000	−0.222946000	0.247123000
C	1.655590000	−0.921754000	0.527091000
C	2.809968000	−0.306756000	−0.117636000
C	3.793986000	0.173064000	−0.624165000
O	−3.050215000	−1.130587000	−0.334578000
H	1.269346000	1.644405000	0.441680000
H	−0.925802000	2.847797000	0.137268000
H	−3.010020000	1.539687000	−0.243503000
H	−0.599435000	−2.052781000	0.033637000
H	1.540272000	−1.953287000	0.185996000
H	1.795136000	−0.945127000	1.613877000
H	4.655390000	0.597579000	−1.087434000

Product # 4

C	−1.448143000	−0.774501000	0.949078000
C	−1.631456000	0.460599000	1.828593000
C	−1.266778000	1.662622000	1.348240000
C	−0.665288000	1.778915000	−0.012035000
C	−0.517982000	0.430806000	−0.767190000
N	−1.672719000	−0.444224000	−0.477145000
C	0.588334000	−0.386951000	−0.101562000
C	0.046975000	−1.096372000	0.895810000
C	1.995388000	−0.316635000	−0.537941000
O	2.375189000	0.345045000	−1.484702000
O	2.800866000	−1.085721000	0.230544000
C	4.945253000	0.075357000	0.501517000
C	4.205748000	−1.096661000	−0.124593000
C	−2.988995000	0.072620000	−0.858158000
C	−4.044642000	−0.910032000	−0.601027000
C	−4.927407000	−1.699848000	−0.368057000
O	−0.314702000	2.846462000	−0.481498000
H	−2.082871000	−1.604158000	1.269895000
H	−2.040233000	0.350139000	2.831049000
H	−1.366612000	2.582741000	1.917196000
H	−0.364941000	0.613505000	−1.832994000
H	0.562484000	−1.748786000	1.588396000
H	6.014963000	−0.001181000	0.280329000
H	4.818917000	0.080802000	1.588084000
H	4.578617000	1.021342000	0.096428000
H	4.292963000	−1.081772000	−1.213426000
H	4.571969000	−2.052847000	0.256315000
H	−2.946985000	0.288860000	−1.933161000
H	−3.261104000	1.019003000	−0.358268000
H	−5.705000000	−2.403871000	−0.180630000

Product # 5

C	0.608749000	0.582260000	0.419466000
C	1.102176000	−0.211794000	1.624367000
C	1.708434000	−1.397391000	1.426360000
C	1.854039000	−1.940938000	0.049438000
C	1.304561000	−1.011181000	−1.071148000
N	1.524272000	0.407238000	−0.731242000
C	−0.215987000	−1.060301000	−1.013244000
C	−0.629599000	−0.124924000	−0.150097000
C	−1.987185000	0.180643000	0.330326000
O	−2.211996000	0.961166000	1.238183000
O	−2.935608000	−0.506709000	−0.341737000
C	−4.892028000	0.942532000	−0.637057000
C	−4.308305000	−0.275154000	0.061990000
C	2.918560000	0.823440000	−0.567125000
C	3.022731000	2.265221000	−0.331088000
C	3.121273000	3.447662000	−0.109457000
O	2.336812000	−3.034353000	−0.188771000
H	0.448621000	1.634797000	0.656981000
H	0.939368000	0.182535000	2.624671000
H	2.075247000	−2.018588000	2.238612000
H	1.744172000	−1.296535000	−2.031278000
H	−0.825294000	−1.781203000	−1.542288000
H	−5.949633000	1.048639000	−0.374011000
H	−4.816509000	0.841676000	−1.723539000
H	−4.369462000	1.850764000	−0.327752000
H	−4.340858000	−0.161472000	1.147978000
H	−4.830427000	−1.191120000	−0.223642000
H	3.439213000	0.569401000	−1.499437000
H	3.451209000	0.294852000	0.243206000
H	3.206284000	4.494317000	0.071406000

Product # 8

C	1.219083000	−2.842613000	−0.777985000
C	−1.057957000	−2.635522000	−0.094333000
N	0.119174000	−3.396575000	−0.101460000
C	−1.076557000	−1.309201000	−0.308841000
C	1.260115000	−1.517776000	−0.946340000
C	0.229011000	−0.548782000	−0.415329000
O	2.127133000	−0.874723000	−1.835898000
C	0.029280000	−4.852797000	−0.011722000
C	1.528524000	0.313279000	−2.134198000
C	0.404792000	0.570626000	−1.436750000
C	−0.355146000	1.810718000	−1.582430000
O	−0.118289000	2.693641000	−2.388580000
O	−1.381738000	1.870095000	−0.692290000
C	−3.265931000	2.944744000	0.308999000
C	−2.187542000	3.067100000	−0.750381000
C	1.148840000	−5.451688000	0.720059000
C	2.076575000	−5.964868000	1.296444000
H	−2.014033000	−0.768094000	−0.323989000
H	0.086716000	−1.966437000	−3.200653000
H	1.949901000	−3.525716000	−1.192291000
H	0.509041000	−0.156667000	0.581371000
H	−0.907706000	−5.095359000	0.499817000
H	−0.029968000	−5.311961000	−1.013068000
H	2.025413000	0.909895000	−2.886403000
H	−3.896160000	3.839451000	0.302920000
H	−2.825768000	2.840597000	1.304820000
H	−3.902868000	2.075296000	0.121848000
H	−2.607735000	3.163855000	−1.756508000
H	−1.543643000	3.936119000	−0.581745000
H	2.892600000	−6.407070000	1.820182000

Product # 9

C	−0.259705000	−1.046552000	−0.224994000
C	−1.553582000	−1.325787000	−0.498612000
N	−2.536300000	−0.357759000	−0.697055000
C	−2.366050000	1.01919	−0.200306000
C	−0.902265000	1.28977	−0.138958000
C	0.09004	0.35625	−0.101748000
O	−0.405919000	2.55795	−0.014678000
C	−3.922294000	−0.787122000	−0.823933000
C	0.94081	2.43141	0.09408
C	1.31377	1.11357	0.04849
C	2.71559	0.68083	0.13945
O	3.6641	1.43391	0.26029
O	2.83737	−0.667191000	0.07417
C	4.11389	−2.686924000	0.05685
C	4.18795	−1.175434000	0.15438
C	−4.641215000	−0.920970000	0.45584
C	−5.214596000	−1.016073000	1.51462
H	0.48153	−1.827171000	−0.141279000
H	−1.895966000	−2.344717000	−0.646437000
H	−2.848804000	1.14972	0.78861
H	−2.876538000	1.70867	−0.889003000
H	−3.941159000	−1.742695000	−1.359436000
H	−4.452548000	−0.064955000	−1.459499000
H	1.51158	3.34077	0.19019
H	5.1206	−3.112604000	0.11241
H	3.6622	−2.996117000	−0.890128000
H	3.51964	−3.103440000	0.87528
H	4.63468	−0.845325000	1.09747
H	4.77865	−0.739264000	−0.657225000
H	−5.718248000	−1.108764000	2.44929

Product # 6

C	0.33516	−1.137479000	−0.169259000
C	1.64893	−1.429496000	−0.410245000
N	2.59966	−0.467889000	−0.614485000
C	2.25685	0.88635	−0.546867000
C	0.96338	1.21655	−0.306868000
C	−0.071972000	0.23223	−0.105042000
O	0.47574	2.47677	−0.233058000
C	4.01263	−0.831552000	−0.753393000
C	4.77358	−0.736878000	0.49676
C	5.40177	−0.656076000	1.52391
C	−0.959060000	2.39535	0.03329
C	−1.249301000	0.91374	0.102
C	−2.586844000	0.43888	0.34544
O	−3.556716000	1.16995	0.50885
O	−2.670695000	−0.930036000	0.38127
C	−4.776768000	−1.601857000	−0.679382000
C	−3.984937000	−1.475427000	0.61439
H	−0.384277000	−1.932928000	−0.033994000
H	2.00641	−2.451798000	−0.458003000
H	3.04981	1.60616	−0.693327000
H	4.46077	−0.181146000	−1.514133000
H	4.05996	−1.851933000	−1.147149000
H	5.9473	−0.583359000	2.43683
H	−1.487520000	2.91597	−0.775521000
H	−1.163240000	2.93357	0.96766
H	−5.738023000	−2.090047000	−0.485226000
H	−4.229781000	−2.199928000	−1.414631000
H	−4.972341000	−0.613732000	−1.102293000
H	−4.511668000	−0.840451000	1.33078
H	−3.802119000	−2.454722000	1.06546

Product # 7

C	−1.822425000	−0.462961000	−0.630678000
C	−1.947799000	−1.779417000	−0.384559000
N	−0.880902000	−2.593888000	−0.010409000
C	0.42018	−2.089871000	−0.153952000
C	0.58544	−0.774784000	−0.337760000
C	−0.531860000	0.2464	−0.277009000
O	1.74615	−0.201434000	−0.841104000
C	−1.112568000	−3.843105000	0.70166
C	−1.208806000	−3.706490000	2.16297
C	−1.287325000	−3.585943000	3.36178
C	1.34113	0.99176	−1.413916000
C	0.05199	1.2971	−1.202400000
C	2.39673	1.72267	−2.150602000
O	3.54958	1.35908	−2.239296000
O	1.89517	2.85221	−2.701738000
C	2.93755	3.17653	−4.897413000
C	2.82748	3.6605	−3.459919000
H	−2.662777000	0.09488	−1.029267000
H	−2.890189000	−2.303175000	−0.517952000
H	1.2248	−2.811939000	−0.236990000
H	−0.621262000	0.69778	0.7323
H	−0.299779000	−4.537113000	0.45243
H	−2.035451000	−4.291956000	0.31399
H	−1.353267000	−3.469404000	4.41896
H	−0.441363000	2.19331	−1.548885000
H	3.58847	3.84894	−5.465990000
H	1.95571	3.15949	−5.379227000
H	3.36747	2.17278	−4.932604000
H	3.79882	3.63866	−2.960460000
H	2.41403	4.67028	−3.406700000

TS #4

C	−2.024449000	0.67658	0.75245
C	−1.556519000	2.0214	1.00447
C	−0.504093000	2.53572	0.32266
C	0.20423	1.7113	−0.656746000
C	−0.444104000	0.42	−0.928341000
N	−1.681706000	0.13727	−0.470396000
C	0.35648	−0.617064000	1.20388
C	−0.669721000	−0.399894000	1.88074
C	1.59453	−1.129311000	0.67288
O	1.70043	−2.208363000	0.11246
O	2.6005	−0.250452000	0.85843
C	3.88159	−0.295808000	−1.237569000
C	3.87509	−0.589452000	0.25541
C	−2.252151000	−1.177879000	−0.843122000
C	−3.701509000	−1.239883000	−0.688787000
C	−4.898072000	−1.327423000	−0.561073000
O	1.24342	2.04389	−1.234581000
H	−3.003940000	0.38589	1.11518
H	−2.047363000	2.58257	1.79407
H	−0.130441000	3.53834	0.50114
H	−0.070778000	−0.166746000	−1.760280000
H	−1.144002000	−0.524571000	2.83734
H	4.90169	−0.382897000	−1.627046000
H	3.50622	0.71251	−1.428393000
H	3.24921	−1.009570000	−1.770663000
H	4.09616	−1.640249000	0.46057
H	4.58887	0.04071	0.79045
H	−1.761791000	−1.943362000	−0.226398000
H	−1.977719000	−1.364281000	−1.885338000
H	−5.956862000	−1.399539000	−0.459464000

TS #5

C	0.75873	0.14962	1.4051
C	1.28174	−1.160670000	1.55156
C	2.39446	−1.566881000	0.86725
C	2.97092	−0.714028000	−0.159618000
C	2.21783	0.55567	−0.365974000
N	1.43816	1.04469	0.65814
C	0.87304	−0.220459000	−1.603504000
C	−0.207006000	−0.561662000	−1.083170000
C	−1.465613000	−1.068497000	−0.633297000
O	−1.644484000	−2.190795000	−0.186794000
O	−2.459359000	−0.146420000	−0.784389000
C	−3.981674000	−0.377277000	1.12747
C	−3.776602000	−0.570932000	−0.367953000
C	1.02406	2.46443	0.65502
C	−0.235021000	2.72883	−0.038296000
C	−1.282126000	2.96937	−0.585827000
O	3.92913	−1.000224000	−0.880685000
H	−0.020051000	0.53418	2.0512
H	0.78214	−1.823670000	2.24973
H	2.8468	−2.538101000	1.03499
H	2.73177	1.30938	−0.954895000
H	1.45907	−0.266801000	−2.507668000
H	−5.013881000	−0.622862000	1.39895
H	−3.788442000	0.66058	1.41556
H	−3.314737000	−1.033947000	1.6912
H	−3.920874000	−1.616374000	−0.650190000
H	−4.458641000	0.05642	−0.947620000
H	1.84167	3.03461	0.20462
H	0.95314	2.78301	1.70076
H	−2.203701000	3.14511	−1.091107000

TS #6

C	−0.683193000	−1.396893000	−0.410911000
C	−1.903594000	−1.486157000	0.22524
N	−2.623337000	−0.356063000	0.46834
C	−2.139856000	0.87161	0.13498
C	−0.929322000	1.02137	−0.539196000
C	−0.171419000	−0.158121000	−0.818086000
O	−0.494054000	2.21977	−0.823635000
C	−3.977666000	−0.465032000	1.05952
C	1.1102	2.3984	−0.327741000
C	1.7392	1.41175	0.14217
C	2.68737	0.54163	0.71416
O	2.8776	0.33565	1.90699
O	3.39039	−0.133835000	−0.266864000
C	5.06694	−1.664694000	−0.985930000
C	4.38642	−1.045059000	0.22199
C	−5.026830000	−0.601753000	0.05215
C	−5.899294000	−0.714822000	−0.773048000
H	−0.139811000	−2.311884000	−0.618204000
H	−2.360046000	−2.422089000	0.51901
H	−2.741176000	1.73203	0.39816
H	0.69038	−0.108731000	−1.465525000
H	−3.974204000	−1.324465000	1.73676
H	−4.142682000	0.4256	1.6735
H	1.21297	3.44857	−0.550189000
H	5.83846	−2.371645000	−0.663692000
H	5.53996	−0.895361000	−1.603197000
H	4.34541	−2.203148000	−1.608227000
H	3.91364	−1.805480000	0.85441
H	5.10015	−0.505485000	0.85416
H	−6.664970000	−0.811468000	−1.508920000

TS #7

C	−1.939153000	2.12427	−0.007136000
C	−2.816499000	1.23253	−0.550298000
N	−2.560028000	−0.116338000	−0.514743000
C	−1.412280000	−0.597902000	0.03711
C	−0.465282000	0.23985	0.6576
C	−0.675926000	1.70418	0.55665
O	0.61195	−0.187271000	1.14976
C	−3.591787000	−1.059594000	−0.987097000
C	−4.569766000	−1.408841000	0.04371
C	−5.382874000	−1.698807000	0.88652
C	1.79475	1.36416	−0.219226000
C	0.81449	2.12696	−0.419345000
C	2.97818	0.58745	0.00103
O	3.79908	0.81831	0.87157
O	3.08163	−0.414112000	−0.910859000
C	3.92077	−2.375073000	0.29352
C	4.21237	−1.297839000	−0.740878000
H	−2.211747000	3.17505	−0.009803000
H	−3.770205000	1.52058	−0.973995000
H	−1.277851000	−1.672170000	0.04559
H	−0.350178000	2.26847	1.43101
H	−3.081163000	−1.956396000	−1.351578000
H	−4.091099000	−0.605921000	−1.849401000
H	−6.092812000	−1.952445000	1.64032
H	0.63719	3.02463	−0.990403000
H	4.76495	−3.070490000	0.3554
H	3.02396	−2.939590000	0.02313
H	3.76666	−1.926548000	1.27725
H	5.08744	−0.707468000	−0.457528000
H	4.37181	−1.726772000	−1.733523000
